# Strategic prioritisation: Three principles for an affordable and essential preparedness package

**DOI:** 10.7189/jogh.13.03052

**Published:** 2023-10-27

**Authors:** Mukesh Chawla, Rocio Schmunis, Mariana Zindel

**Affiliations:** The World Bank, Washington DC, USA

## THE CONTEXT

Despite decades of warnings and years of international planning, the coronavirus disease 2019 (COVID-19) pandemic caught the world off guard, resulting in unprecedented human and economic losses [1]. The pandemic exposed profound weaknesses in the world’s infectious disease detection and response capabilities, which have stubbornly persisted despite causing enormous harm during prior outbreaks. On 5 May 2023, the World Health Organization (WHO) downgraded the COVID-19 pandemic and declared it no longer qualified as a public health emergency. At the same time, it reminded the world of the high risk of new variants causing massive surges in cases and deaths and cautioned countries against letting their guard down [2]. While the worst of the pandemic may be over, whether we experience the same levels of death and devastation again will depend on the investments in strengthening preparedness we make now.

Estimates of new investments that countries and global institutions would need to address the existing gaps in pandemic prevention, detection, and response capabilities vary between US$15 and US$50 billion, depending upon definitions and methodologies used in computing these numbers. The Group of Twenty (G20) High-Level Independent Panel suggests a minimum additional annual country-level investment of US$10 billion, along with an extra 5 billion to strengthen WHO and other existing institutions, to plug gaps in surveillance, resilient health systems, medical countermeasures, and global governance [3]. In contrast, McKinsey and Company recommends allocating US$85 to US$130 billion over two years, followed by annual investments of US$20 to US$50 billion, to fund five preparedness areas: building “always-on” response systems, strengthening mechanisms for detecting infectious diseases, integrating efforts to prevent outbreaks, developing health care systems that can handle surges while maintaining the provision of essential services, and accelerating research and development for diagnostics, therapeutics, and vaccines [4]. A recent analysis estimates annual funding needs of US$31.1 billion to fund five preparedness sub-systems proposed by WHO: collaborative surveillance, community protection, safe and scalable care, access to countermeasures, and emergency coordination [5,6]. Eaneff et al. projects a total requirement of US$124 billion over five years to progress towards the benchmarks identified by WHO’s Joint External Evaluation (JEE) [7]. Notwithstanding the differences in methodology and scope, these estimates suggest that modest investments of US$4 to US$7 per person per year can significantly enhance preparedness for future pandemics. Yet, many countries will not be able to afford these recommended investments, especially the 51 low-income countries in which current general government spending on health is less than US$50 per person per year [8]. These countries have huge unmet proximate needs in the health sector and will find it challenging to allocate additional resources to prevention and public health until the urgent gaps in curative care have been met. Earmarked donor assistance offers only a partial solution since most of the expenditure on preparedness strengthening is recurrent in nature and can only be sustained if supported by domestic resources. Until income levels increase sufficiently, most low-income countries will likely be able to mobilise only a fraction of the new financing needed for strengthening preparedness.

In the face of these limitations, low-income countries would need to ration resources to a few areas of preparedness that warrant prioritised investment. Confronted with a multitude of considerations, including the 19 technical areas identified in the JEE, the five preparedness sub-systems proposed by WHO [6], as well as the pressing need to accelerate research and development for diagnostics, therapeutics, and vaccines, these countries would need to establish actionable criteria to strategically select from the extensive array of preparedness measures recommended by global agencies and technical experts. Informal approaches employed thus far, primarily relying on expert advice and prioritising pre-approved and budgeted new or ongoing activities, and areas where donor assistance is readily available, have failed to address the urgent gaps and have resulted in minimal investments in strengthening preparedness [9]. This article proposes a practical and evidence-based way of identifying an essential and affordable set of interventions to establish a high level of preparedness against major disease outbreaks.

## PRINCIPLES OF PRIORITISATION

**Figure 1:**
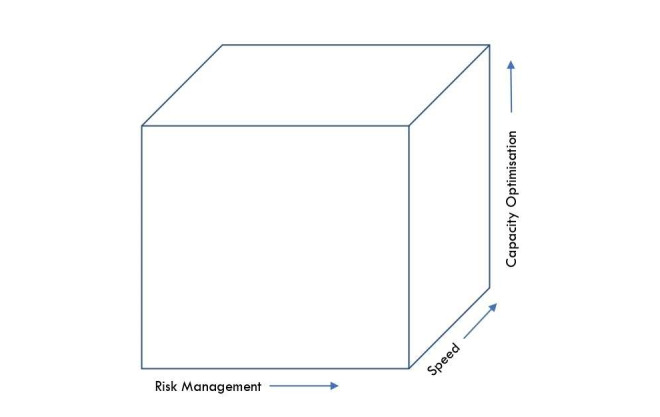
The prioritisation cube.

We propose three principles of prioritisation aimed at identifying and allocating resources to a range of interventions that collectively enhance preparedness at a high level: risk management, speed maximisation, and capacity optimisation. Collectively, these principles form the prioritisation cube (**Figure 1**), which provides a holistic framework for understanding prioritisation and guiding decision-making. Through their interconnected yet distinct perspectives, these principles consider many factors when establishing priorities. The following sections elaborate on these principles.

### Manage risk

We suggest risk management as a fundamental basis for prioritisation. Public health risks emerge from the interplay between biological, technological, societal, and natural hazards and the communities they affect. Failure to manage these risks effectively can result in profound health and economic ramifications. A risk-based approach to prioritisation suggests that countries allocate their limited resources first and foremost to strengthen detection and response capacities to address disease outbreaks to which they are most vulnerable and which are likely to have the most severe health and economic consequences. To implement this approach, countries would need to conduct a comprehensive assessment of their public health risks and vulnerabilities. This assessment would serve multiple purposes, including guiding actions, informing planning, and providing evidence for developing strategies and policies to enhance prevention, preparedness, response, and recovery efforts. Establishing a profile with details about risks and coping capacities would enable countries to anticipate threats, enhance readiness, and foster a more effective emergency response. Risk assessment is recognised as a fundamental element of the International Health Regulations and the Sendai Framework for Disaster Risk Reduction, underscoring its importance in promoting comprehensive and strategic preparedness measures.

A valuable resource for countries adopting a risk-management approach is WHO’s Strategic Tool for Assessing Risks (STAR) [10]. This tool offers a comprehensive and user-friendly toolkit designed to enable national and subnational governments to rapidly conduct a strategic and evidence-based assessment of public health risks. STAR assists in identifying country-specific hazards and describing the most likely scenarios that would necessitate the activation of a national response. It also facilitates the assessment of the likelihood of risk occurrence, including considerations of severity and seasonality, as well as estimating the impact of the risk on the country. Utilising the assessed risk ranking, STAR further guides initial short-term planning and prioritisation of health emergency preparedness and disaster risk management activities. By leveraging this tool, countries can enhance their ability to make informed decisions, allocate resources effectively, and implement appropriate measures to address identified risks and vulnerabilities.

### Maximise speed

Another fundamental basis of prioritisation is the maximisation of speed of detection and response. Speed is of the essence in responding to a viral outbreak to contain the spread, implement preventive measures, provide effective treatment, monitor the situation, raise public awareness, and mitigate the economic impact. Acting quickly increases the chances of successfully managing and controlling the outbreak, safeguarding public health and well-being. To implement this approach, countries would need to strengthen national and subnational capacities to detect and respond to disease outbreaks quickly. Frieden et al. propose an effective and practical seven-one-seven metric, which suggests that countries should aim to, first, detect a suspected infectious disease outbreak within seven days, second, notify public health authorities within one day to initiate an investigation, and third, complete an initial response within seven days [11]. The seven-one-seven approach can help countries establish agile and accountable systems and use the timeliness metric and real-world events to monitor response during a health crisis and course correct as needed [12].

In a five-country retrospective review of public health events and associated milestone dates according to the seven-one-seven metric, Bochner and colleagues identify 129 conditions, or bottlenecks, that caused delays in actions and 162 conditions, or enablers, that facilitated prompt actions. To assess the alignment between these operational capabilities and the technical capacities in the JEE tool, they map each bottleneck and enabler to JEE indicators. Of 291 total bottlenecks and enablers identified, 248 (85%) were successfully linked to existing JEE indicators. These indicators primarily focused on reporting channels, case management procedures (including the implementation of case definitions and standard operating procedures (SOPs) at health facilities), availability of human resources, laboratory diagnostic capacity, and funding availability for timely response to health emergencies. However, 40 (12%) bottlenecks or enablers did not align with any JEE indicators, especially those related to access in conflict or remote settings, prioritisation issues associated with the COVID-19 pandemic, technological obstacles such as lack of mobile network coverage, and low community knowledge or trust in the public health system. In three cases, there was insufficient information to assign a specific JEE indicator [13].

We draw upon the findings reported in Bochner et al. and identify eleven broad capacities that we believe countries would need to prioritise to maximise speed of detection and response: strong event-based surveillance and case investigation capacity, available and functioning at the village, community, district and national level; advanced capacity in select national public health laboratories, equipped to diagnose pathogens of interest; functioning specimen referral and transport system; rapid reporting mechanism to a public health authority responsible for action; strengthened relationships with community leaders, including build-up of trust and engagement; rapid alert and response coordination, including operations support and logistics; diversified supply-chains and strategic stockpiles; established processes and procedures for case management, infection prevention and control, and access to medical countermeasures; stable, robust and functioning border health control systems; community knowledge and trust in the public health system; and availability of flexible funds for deployment of teams and availability of countermeasures at subnational levels. By investing in these capacities, countries can enhance their ability to swiftly identify, investigate, and respond to disease outbreaks and minimise their impact on public health and socioeconomic well-being.

Countries with weak health systems, characterised by insufficient investments in public health and shortages of health care professionals, may need to mobilise considerable financing to hire and train health care workers, especially at community and district levels. On the other hand, countries with already robust public health structures would primarily incur additional operational and programmatic costs to maintain a high state of readiness and ensure rapid deployment of response mechanisms in line with the seven-one-seven approach. These costs would be associated with sustaining the existing systems, optimising coordination and communication channels, and continuously enhancing preparedness measures. Regardless of the starting point, both types of countries would need to make strategic investments to strengthen elements of the health systems, enhance capacity, and effectively implement the seven-one-seven approach to better prepare for and respond to disease outbreaks.

### Optimise capacity

Our third fundamental basis of prioritisation is capacity optimisation. The COVID-19 pandemic exemplified how even countries with ample resources and robust systems struggled to cope with the sudden surge in infections, fatalities, and economic upheaval. Consequently, their response efforts fell significantly short of their potential. This predicament becomes particularly critical for low-income countries, which already face inherent system limitations. To effectively tackle a major disease outbreak, countries must establish structures and policies that enable them to optimise their limited resources and respond promptly. Embracing a capacity-based approach to prioritisation ensures that crisis response efforts are maximally effective by aligning actions with existing capabilities, guaranteeing feasibility and attainability. By prioritising capacity-based strategies, response efforts become realistic, adaptable to local contexts, and optimised for the specific challenges at hand. To adopt this approach, we recommend that countries proactively establish rules, regulations, protocols, and SOPs that offer explicit guidance on the execution of specific tasks and activities during a health crisis. By developing these frameworks ahead of time, nations can ensure that response measures make optimal use of their available capacity and resources while upholding standards of efficiency, effectiveness, and safety.

Developing a comprehensive checklist that encompasses the essential elements of a robust preparedness and response system is crucial for optimising capacity. This checklist could include guidelines for key aspects such as case identification and reporting, infection prevention and control, case management, laboratory testing and diagnostics, risk communication and public awareness, travel and border health, quarantine and isolation, surveillance and data reporting, resource management and logistics, as well as monitoring and evaluation. These guidelines are designed to be implemented within the country’s existing capacity or by leveraging readily available capacity outside the country.

Regular updates and refinements of these guidelines are essential, drawing upon lessons learned from previous outbreaks and emerging evidence, as well as the country’s own expanding capacity. This iterative process ensures the ongoing relevance and effectiveness of the guidelines in guiding response efforts. By establishing comprehensive and up-to-date protocols, countries can strengthen their preparedness and response systems, enabling them to respond promptly and effectively to future health crises.

## THE ABSOLUTE MINIMUM PREPAREDNESS PACKAGE

Prioritisation based on the principles of managing risks, maximising speed, and optimising capacity would help low-income countries finance a set of basic and essential measures and capabilities they must have in place to detect, respond to, and mitigate the spread of a pandemic rapidly and effectively. Bundled together to constitute an Absolute Minimum Preparedness Package (AMPP), these essential measures are interventions strategically selected from the five preparedness sub-systems proposed by WHO [6] that ensure that resource-constrained low-income countries are always prepared to handle most major disease outbreaks. Until countries have the necessary resources to fulfil these sub-systems' requirements, implementing the more affordable AMPP provides essential readiness assurance for most major disease outbreaks. **Table 1** presents the key elements of this package.

**Table 1:** Key elements of the Absolute Minimum Preparedness Package

Prioritisation principle	Essential measures
Manage risks	1. Conduct a comprehensive assessment of public health risks and vulnerabilities.
Maximise speed	2. Develop strong event-based surveillance and case investigation capacity at the village, community, district, and national level.
	3. Develop advanced capacity in select national public health laboratories, well-equipped to diagnose pathogens of interest.
	4. Develop a functioning specimen referral and transport system.
	5. Establish a mechanism to immediately report an outbreak to the responsible public health authority.
	6. Develop strong trust and relationships and continuous engagement with community leaders.
	7. Develop a strong response coordination mechanism, including operations support and logistics.
	8. Establish diversified and efficient supply-chain management systems to ensure timely delivery during a health emergency; maintain adequate stockpiles of essential medical supplies.
	9. Develop processes and procedures for case management, infection prevention and control, and access to medical countermeasures.
	10. Establish stable and functioning border health control systems.
	11. Deepen community knowledge and trust in the public health system.
	12. Ensure the availability of flexible funds for the deployment of teams and the availability of countermeasures at subnational levels.
**Optimise capacity**	13. Develop rules, regulations, protocols, and standard operating procedures with clear guidance on how specific tasks and activities would be executed during a health crisis.

## CONCLUSION

This article presents three principles of prioritisation and outlines a set of essential interventions aimed at assisting low-income countries in maintaining a strong baseline level of preparedness and response capacity for major disease outbreaks. To provide an example, we have evaluated the cost of these interventions for Sierra Leone based on their National Action Plan for Health Security (NAPHS) [14]. The estimated one-time start-up and capital costs amount to US$3.04 million, while the annual recurrent costs total US$7.26 million. These figures are significantly lower than the estimated costs of all JEE activities, which stand at US$47.41 million for one-time expenses and US$58.26 million for annual recurrent costs. On a per capita basis, the annual expenses for the recurrent annual portion of the prioritised AMPP would be only US$0.87 per person per year, well below the estimated requirement of US$4 to US$7 per person per year. We anticipate that the cost of the AMPP will be significantly lower than the NAPHS in other low-income countries as well. Consequently, we consider the AMPP to be an affordable interim investment strategy for establishing essential baseline preparedness in most nations. This approach provides a realistic overview to policymakers, showcasing achievable outcomes even in financially constrained circumstances.
